# Welsh Education

**Published:** 1916-07-01

**Authors:** 


### WELSH EDUCATION.

### Commissioners' Visit to Cardiff.

Lord Haldane and the other members of the Royal Commission paid an informal visit to the University College of South Wales and Monmouthshire on Wednesday last week, and inspected its buildings in the morning. They were entertained to luncheon by Lord Aberdare, the president of the college, and in the afternoon they visited King Edward VII.'s Hospital and the Welsh National Medical School Buildings, now in course of erection in the Newport Road. The following day they visited the New Technical College in the Catheys Park, and were entertained to tea in the City Hall by the Lord Mayor (Dr. Smith). Although not in the official programme, they visited the Mental Hospital at Whitchurch, where they were solely concerned with the laboratories provided for giving modern instruction and conducting research on mental disorders.

Although all the proceedings were quite informal, it is true to say, from the expressions of the Commissioners themselves, that the two days' visit to Cardiff was a pleasant one, and that they were much impressed by the facilities afforded in the various buildings in the city for higher education. They were particularly struck with the beautiful wards and the up-to-date character of King Edward VII.'s Hospital, the facilities afforded for teaching, its pathological institute, and the new physiological buildings of the Medical School. Of course, the main purpose of this visit was not so much to take evidence—which will be done when the Commission sits in London during this month—as to have personal conversations with prominent educationists who are interested in the reorganisation of the University of Wales, and in the establishment and development of the Welsh National School of Medicine.

During the course of the proceedings, Lord Haldane said that the Commission was in Wales on a voyage of discovery, and they were groping about amid unlimited possibilities in search of information with regard to the best means of dealing with university education in the Principality. He discussed at length the advantages and disadvantages of a federated university, for the Commission at present had an open mind on the question. It was quite possible, he averred, that there might be a need for the establishment of some type of university which would appeal more unanimously than the one now in existence to Welsh sentiment as a whole.

The idea of a separate university for South Wales and Monmouthshire at Cardiff was perhaps the outstanding topic during the visit, and it is generally felt that the Cardiff College will not become a part of the local life and interests of the city until it is severed from the spectral body called the University of Wales.

Sir William Osler said that the Commission was greatly pleased with its visit, and saw no difficulty in the establishment at Cardiff of a great school of medicine.

